# Cutaneous sensitivity in unilateral trans-tibial amputees

**DOI:** 10.1371/journal.pone.0197557

**Published:** 2018-06-01

**Authors:** Cale A. Templeton, Nicholas D. J. Strzalkowski, Patti Galvin, Leah R. Bent

**Affiliations:** 1 Department of Human Health and Nutritional Sciences, University of Guelph, Guelph, Ontario, Canada; 2 Department of Clinical Neuroscience, University of Calgary, Calgary, Alberta, Canada; 3 Wellington Ortho and Rehab, Guelph, Ontario, Canada; University of Chicago, UNITED STATES

## Abstract

**Aim:**

To examine tactile sensitivity in the leg and foot sole of below-knee amputees (diabetic n = 3, traumatic n = 1), and healthy control subjects (n = 4), and examine the association between sensation and balance.

**Method:**

Vibration perception threshold (VPT; 3, 40, 250Hz) and monofilaments (MF) were used to examine vibration and light touch sensitivity on the intact limb, residual limb, and homologous locations on controls. A functional reach test was performed to assess functional balance.

**Results:**

Tactile sensitivity was lower for diabetic amputee subjects compared to age matched controls for both VPT and MF; which was expected due to presence of diabetic peripheral neuropathy. In contrast, the traumatic amputee participant showed increased sensitivity for VPT at 40Hz and 250Hz vibration in both the intact and residual limbs compared to controls. Amputees with lower tactile sensitivity had shorter reach distances compared to those with higher sensitivity.

**Conclusion:**

Changes in tactile sensitivity in the residual limb of trans-tibial amputees may have implications for the interaction between the amputee and the prosthetic device. The decreased skin sensitivity observed in the residual limb of subjects with diabetes is of concern as changes in skin sensitivity may be important in 1) identification/prevention of excessive pressure and 2) for functional stability. Interestingly, we saw increased residual limb skin sensitivity in the individual with the traumatic amputation. Although not measured directly in the present study, this increase in tactile sensitivity may be related to cortical reorganisation, which is known to occur following amputation, and would support similar findings observed in upper limb amputees.

## Introduction

Sensory feedback from the glabrous skin on the foot soles has been shown to contribute to the maintenance of standing balance and control of gait [1]. When foot sole cutaneous feedback is reduced experimentally through cooling or topical anaesthetic, increased centre of pressure (CoP) excursions are observed during quiet stance [2], and gait is altered [3]. Reductions in afferent feedback as a consequence of age [4], diabetes [5], and Parkinson’s disease [6] have also been shown to contribute to a decline in balance and altered gait patterns. Following lower limb amputation, the cutaneous tactile information from the amputated foot sole is lost which may present a considerable challenge to an amputees’ recovery of balance during rehabilitation.

At the onset of rehabilitation, amputees exhibit an increased dependence on vision during upright stance [7]. Interestingly, this increased dependence on vision returns to baseline following eight months of balance training [7], and it has been suggested that an up-regulation of other sensory systems, such as proprioceptive feedback from the intact skin may help compensate for the loss of sensory feedback caused by the amputation [7, 8]. The skin that associates with the prosthetic is of particular interest, as this area signals information regarding weight bearing and pressure within the prosthetic, similar to the role of the foot sole in non-amputated individuals. Studies which have caused deafferentation via ischemic block in animals [9, 10] and humans [11] or cutaneous anesthesia [12] have shown that cortical reorganisation can occur within minutes after sensation is lost, and human studies have shown deafferentation of the upper limb led to increased sensory acuity and sensitivity in the neighbouring intact areas [12–14]. Therefore, it is reasonable that cutaneous afferent feedback originating within the prosthetic may become up-regulated (given more functional weighting) as it takes on the task of weight bearing, and sensory role of the amputated foot. This up-regulation of residual limb skin feedback could exhibit as reduced perception or two-point discrimination thresholds. These changes are thought to be due to reorganisation within the central nervous system, termed cortical reorganisation [15, 16]. Following amputation, areas of the somatosensory cortex which previously corresponded to the amputated limb become responsive to stimulation of body regions corresponding to the neighbouring cortical areas [16, 17]. While the exact link between altered cortical representations and changes in tactile sensation has yet to be identified, these cortical changes may represent an adaptation to the loss of sensory feedback following amputation [15, 18].

To date, research examining cutaneous sensation in lower limb amputees has largely used qualitative measures (i.e. classifying sensation as “normal” or “impaired”) rather than capitalizing on controlled experimental designs [18, 19]. In addition, it is critical to consider the nature of the amputation and include control limb comparisons. Individuals who have undergone amputation due to diabetes often have peripheral neuropathies which affect tactile sensation bilaterally; thus, amputees with diabetes should be analysed separately from traumatic amputees, which has not been the case in all previous work [20]. Furthermore, MRI scans in humans have shown that cortical reorganisation may be occurring in both hemispheres of the brain following amputation, which suggests that bilateral changes in tactile sensitivity may be occurring [21]. Therefore, it is important to compare skin sensation on the amputated limb to healthy, non-amputated control subjects rather than to homologous locations on the intact leg of amputee subjects. Finally, despite the frequent use of quantitative vibration perception threshold (VPT) testing in the study of cutaneous sensation in the healthy population [22, 23], it has yet to be used in amputees. VPT testing across different frequencies provides the advantage of varying the activation levels of different classes of cutaneous afferents [slowly adapting type I and II (SAI and SAII) and fast adapting type I and II (FAI and FAII)]. SAI afferents relay information about pressure application to the skin [22], while SAII respond to skin stretch which occurs during joint movement [24]. FAI and FAII afferents provide information about velocity of skin indentation, and in the foot sole, are important for signalling and responding to dynamic events such as slips and trips [25]. Each of these afferent classes are uniquely sensitive to different frequency ranges, which gives VPT testing the unique ability to selectively activate different sensory channels [26, 27]. This information may provide insight into how feedback from specific afferent classes adapts (upregulated or downregulated) following amputation.

The purpose of this study was to examine changes in tactile perception thresholds (sensitivity) of light touch and vibration in lower limb amputees and to relate these thresholds to functional balance. Monofilaments were used to assess light touch, and VPT was tested at three frequencies (3, 40, 250Hz) to selectively target different populations of cutaneous receptors. Functional balance (functional reach) was related to tactile sensitivity by correlating maximum reach, and maximum excursions in CoP with changes in perception threshold at sites in contact with the prosthetic. It was hypothesized that traumatic amputees would show increased sensitivity of the skin around the amputation compared to control subjects, while amputees with diabetes would show decreased sensitivity compared to controls. Furthermore, we predicted that higher sensitivity scores at prosthetic sites would relate to better performance on balance tests.

### Subjects

Five amputees (all male) with unilateral trans-tibial amputations were recruited through a rehabilitation medical practice located in Guelph, Ontario. Subjects were initially screened and excluded if the time since amputation was less than 12 months or the residual limb was less than 15cm long measured from the popliteal crease. The amputees were classified based on the cause of amputation as either diabetic (n = 3, mean age = 59.3±11.5 years, range 48–71 years), or traumatic (n = 1, age 52). Four diabetic amputees were tested however one subject was excluded from data analyses due to positive responses on all catch trials. All of the four analysed amputee subjects were at least 2 years post amputation; 2 of the diabetic amputees were 2 years post and one was 20 years post. The traumatic amputee was 18 years post amputation. In the diabetic group, two amputations were due to diabetic ulcer and one was due to osteomyelitis associated with diabetes. The cause for the amputation in the case of the individual with the traumatic amputation was due to a motorcycle accident 18 years prior to study participation. Age and sex matched control subjects (n = 4, all male, mean age = 54.3±6.6 years, range 46–62) were selected from the University of Guelph population. None of the control subjects had a history of neurologic, visual or vestibular disorders, or systemic disease. Phantom limb sensations were described qualitatively using a subject questionnaire. Frequency of phantom limb sensations were described on a five-point scale from Never to Very Often (daily or multiple times per day). All subjects provided written informed consent prior to participation. The study protocol was approved by the Research Ethics Board at the University of Guelph.

## Materials and methods

### Block 1: Tactile sensation testing

**Fig 1:**
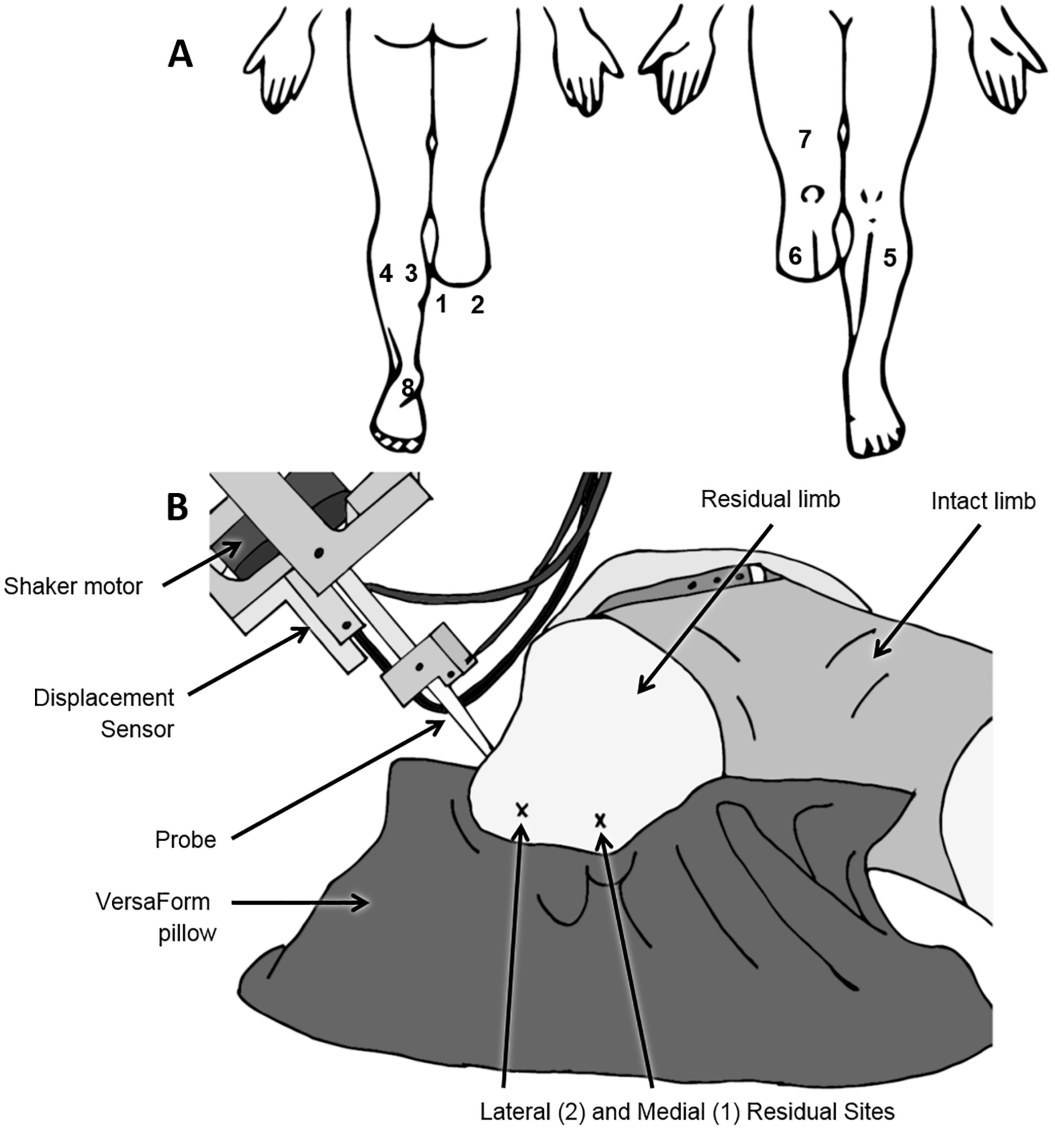
A) Skin sites tested: medial residual (1), lateral residual (2), medial calf (3), lateral calf (4), intact tibia (5), amputated tibia (6), quadriceps (7), heel (8). B) Image of mini-shaker experimental set-up.

Tactile sensation testing included VPT and light touch monofilament thresholds (MF). VPT was always performed first, as this was the most attention demanding portion of the protocol. Eight skin sites were examined in both control and amputee subjects (Fig 1A). All non-glabrous skin sites (i.e. all except heel) were lightly shaved prior to testing to remove any hair. On the amputated limb, two sites on the base of the residual limb were selected. The exact locations of these sites varied slightly, but were never directly on bone or scar tissue and were always on the back skin flap. Of the two residual limb sites, one site was located medially (Site 1—medial residual) and one laterally (Site 2—lateral residual). The distance from the popliteal fossa to the medial and lateral residual was measured and used to determine two homologous sites on the intact calf (Site 3—medial calf and Site 4—lateral calf). These sites were 10-12cm distal to the popliteal crease on average. For controls, calf sites on both legs were measured 10-12cm from the popliteal crease. The remaining sites were the same for amputees and controls. The tibial sites (Sites 5 and 6) were measured 5cm distal to the lateral tibia condyle on both legs. The quadriceps site (Site 7) was located 10cm proximal to the patella in the midline of the leg; this was measured on the amputated limb for amputee subjects and the right limb for control subjects. The heel site (Site 8) was in the middle of the heel, 3cm anterior to the posterior border of the dominant foot for control subjects (right in all cases) and the intact foot of amputee subjects. The order that sites were tested was randomized for both VPT and monofilament testing. Subjects lay on an adjustable treatment bed in a supine or prone position depending on the site being tested, and their test leg was supported with VersaForm pillows (Fig 1B).

#### Vibration sensitivity

VPT was measured at three frequencies (3, 40, 250 Hz) at each test site. Each frequency was selected to provide a different quality of afferent feedback. Although the ability to isolate different afferent classes in the foot sole with vibration is unlikely [27], each afferent class does preferentially responds to different frequencies: SAI afferents (3 Hz), FAI afferents (40 Hz) and FAII afferents (250 Hz) [26, 27]. Subjects had their eyes closed and listened to brown noise during vibration testing. Sinusoidal displacements were delivered using the moving coil of an electromagnetic vibrator (mini-shaker type 4810, Brüel and Kjaer, Naerum, Denmark). The vibrator was mounted on a movable arm with a gimbal socket, allowing the probe to be positioned perpendicular to the skin (Fig 1B). VPT (μm) was measured using a binary search method, details of which have been published previously [4, 28, 29]. Briefly, vibration stimuli were applied as 2-second bursts with ~3-seconds given in between stimuli. Subjects used a trigger to indicate when they perceived a vibration burst. Perceived stimuli resulted in a decrease in vibration amplitude while non perceived stimuli resulted in an increase in vibration amplitude. Trials consisted of 11 vibration bursts, and VPT was the smallest perceived amplitude. Three trials were performed at each frequency and at each site. At each site VPT was the average of the three trials. All three frequencies were examined at one site in random order before examining the next site. VPT testing took 12 mins per site. Sinusoidal displacements were superimposed on an initial indentation produced by a controlled preload force of 1N for 3Hz and 40Hz and 2N for 250Hz. Force (31 load cell, Honeywell, MN, USA) and acceleration (model 2221D, Endevco, CA, USA) data were digitized at 1000Hz using Spike software (CED 1401, Cambridge Electronic Design Limited, Cambride, UK). Displacement of the probe was sampled at 1000Hz and measured using a displacement sensor (model RGH24Z, Renishaw, Glouscestershire, UK) which was able to resolve changes in displacement of 0.5μm.

#### Monofilament sensitivity

Light touch perception was assessed using Semmes-Weinstien Monofilaments (MF) (North Coast Medical Inc., Gilroy, CA). MF threshold was determined using the Dyck 4-2-1 method [30]. Subjects were instructed to provide a “yes” response whenever a stimulus was perceived with 90% confidence. Catch trials (no stimulus applied) were presented randomly during the testing of each skin site (at least 1 catch trial was performed at each site, and one was performed every 8–10 stimulations). Data were discarded if a subject gave a “yes” response on >50% of catch trials. Threshold was determined as the lowest monofilament which could correctly be perceived 75% of the time.

### Block 2: Balance testing functional reach

The functional reach task [31] was used to assess standing balance. Subjects stood on a force plate (model OR6-6, AMTI, Watertown MA) with their feet shoulder width apart. They were instructed to reach as far forward as comfortable without letting their heels come off the plate, and then return to the standing position immediately. Amputees were instructed to reach with the arm contralateral to their amputated limb, while control subjects reached with a self-selected arm. Amputee subjects wore their prosthetic device and associated footwear, and control subjects wore shoes during the reach. Forces and moments were sampled at 100Hz (CED 1401MKI, Cambridge Electronic Design, Cambridge UK) and video was recorded in the sagittal plane at a sampling rate of 30Hz (PowerShot XI, Canon, Japan). Maximum CoP excursion and reach distance were measured. Two separate reaches were recorded for each participant and averaged.

### Data analysis and statistics

VPT were measured in peak-to-peak displacement (μm) of the probe and MF were measured in grams of force (g). Maximum anteroposterior CoP excursion and reach distance (cm) were measured for the functional reach task. Correlations were run between VPT and maximum CoP as well as between VPT and maximum reach distance. For a subset of VPT and MF tests, some subjects were unable to perceive the largest stimulus, resulting in a ceiling effect (see Results, Table 1). Where statistical comparisons were not possible due to low subject size or ceiling effects, trends were described. This was the case for 3 Hz and 250 Hz data for amputees with diabetes, and 250 Hz data for control subjects (Table 1). Data were analysed for normality (Shapiro-Wilk test) and homogeneity of variance (Levene’s test and Bartlett’s test) and were subsequently analysed using parametric or non-parametric methods where appropriate. When comparisons are made between amputees and controls, the dominant leg (right) of controls was compared with the intact leg of amputees, and the non-dominant leg (left) of controls was compared with the amputated limb. Due to non-homogeneity of variance, a Kruskal Wallis test was used to assess differences between the control and amputee groups at 40 Hz VPT with significance set at chi squared <0.05. The Kuskal Wallis test compares all groups and shows that statistically significant differences exist, but is not able to show which comparisons are statistically different (as an ANOVA would). To test site differences at each frequency, individual one-way repeated measures ANOVAs were run within groups (3 Hz control, 40 Hz control and 40 Hz diabetics) with significance set at p<0.05.

**Table 1:** Mean (SD) probe displacement (μm) during vibration perception thresholds (VPT) across test sites and groups. Monofilament threshold data presented in last row (g). Shaded cells indicate at least one subject in that group illustrated ceiling effects. No standard deviations are reported for Traumatic group due to low n (n = 1).

		Amp / Left Tibia	Intact / Right Tibia	Heel	Quad	Lateral Residual	Medial Residual	Lateral Calf	Medial Calf
3 Hz	Control	426.54 (±127.80)	440.85 (±23.74)	279.13 (±33.82)	386.71 (±58.23)	506.17 (±92.68)	583.99 (±113.60)	524.65 (±32.18)	627.13 (±166.40)
	Diabetic	850.19 (±272.50)	720.60 (±501.60)	1198.50 (±278.40)	436.92 (±229.50)	998.94 (±173.30)	917.00 (±394.20)	1030.97 (±275.40)	1052.67 (±615.10)
	Traumatic	670.83	704.42	351.58	559.08	236.75	334.00	615.33	459.47
40 Hz	Control	110.71 (±32.59)	129.56 (±46.86)	56.06 (±18.64)	175.38 (±31.76)	124.85 (±68.18)	123.77 (±34.43)	86.98 (±31.42)	120.17 (±25.71)
	Diabetic	354.14 (±200.20)	249.47 (±186.50)	614.42 (±125.70)	180.14 (±81.07)	265.14 (±102.30)	384.03 (±103.50)	304.81 (±228.50)	178.28 (±136.40)
	Traumatic	12.92	30.00	14.17	24.83	49.17	24.50	47.25	38.08
250 Hz	Control	7.90 (±5.15)	16.71 (±9.35)	4.17 (±2.50)	20.38 (±6.98)	30.50 (±4.15)	28.23 (±4.73)	22.25 (±10.40)	34.00 (±2.67)
	Diabetic	34.26 (±6.49)	25.69 (±14.80)	31.64 (±1.83)	32.42 (±1.70)	32.92 (±1.88)	36.42 (±4.13)	29.83 (±5.67)	26.22 (±6.28)
	Traumatic	2.92	7.25	3.33	4.67	13.83	7.58	11.33	19.00
MF (g)	Control	1.75 (±0.50)	2.00 (±1.40)	4.85 (±3.64)	1.10 (±0.66)	5.25 (±6.60)	1.35 (±0.47)	2.15 (±2.50)	2.20 (±2.50)
	Diabetic	100.00 (±69.20)	42.30 (±51.50)	300.00 (±0.00)	1.00 (±0.00)	122.00 (±156.00)	108.00 (±166.00)	64.00 (±100.00)	62.00 (±101.00)
	Traumatic	1.00	8.00	100.00	0.60	1.00	0.60	4.00	1.40

## Results

Data from one traumatic amputee (male, age 52) and three diabetic amputees (n = 3, all male, mean age 59.3±11.5 years, range 48–71) were used for analysis. The main finding of the present study were the different VPTs between diabetic and traumatic amputees and age matched controls. Specifically, diabetic amputee subjects had decreased cutaneous sensitivity (i.e. increased perception thresholds) at 40 Hz (chi square<0.0001), while the traumatic amputee illustrated greater sensitivity (i.e. decreased perception thresholds), at 40 Hz and 250 Hz vibration at all sites compared to age matched controls.

### Ceiling effects

The shaker apparatus used for VPT restricted the maximum displacement of the probe for a given frequency (1500μm for 3Hz, 2000μm for 40Hz and 35μm for 250Hz). Pilot testing in young healthy subjects (age 20–35) classified these probe displacements consistently as suprathreshold for perception. However, in the current work some subjects were unable to detect the maximum displacement, and therefore true perceptual threshold could not be determined. In these cases, the maximum displacement delivered to that subject was identified as threshold; likely resulting in an underestimation of the true threshold. Data in which ceiling effects were observed were not analysed statistically and are purely descriptive (Table 1). Data from the 40 Hz condition exhibited no ceiling effects across any of the groups and sites and therefore was subjected to statistical analysis.

### Cutaneous sensation

#### Amputees with diabetes

**Fig 2:**
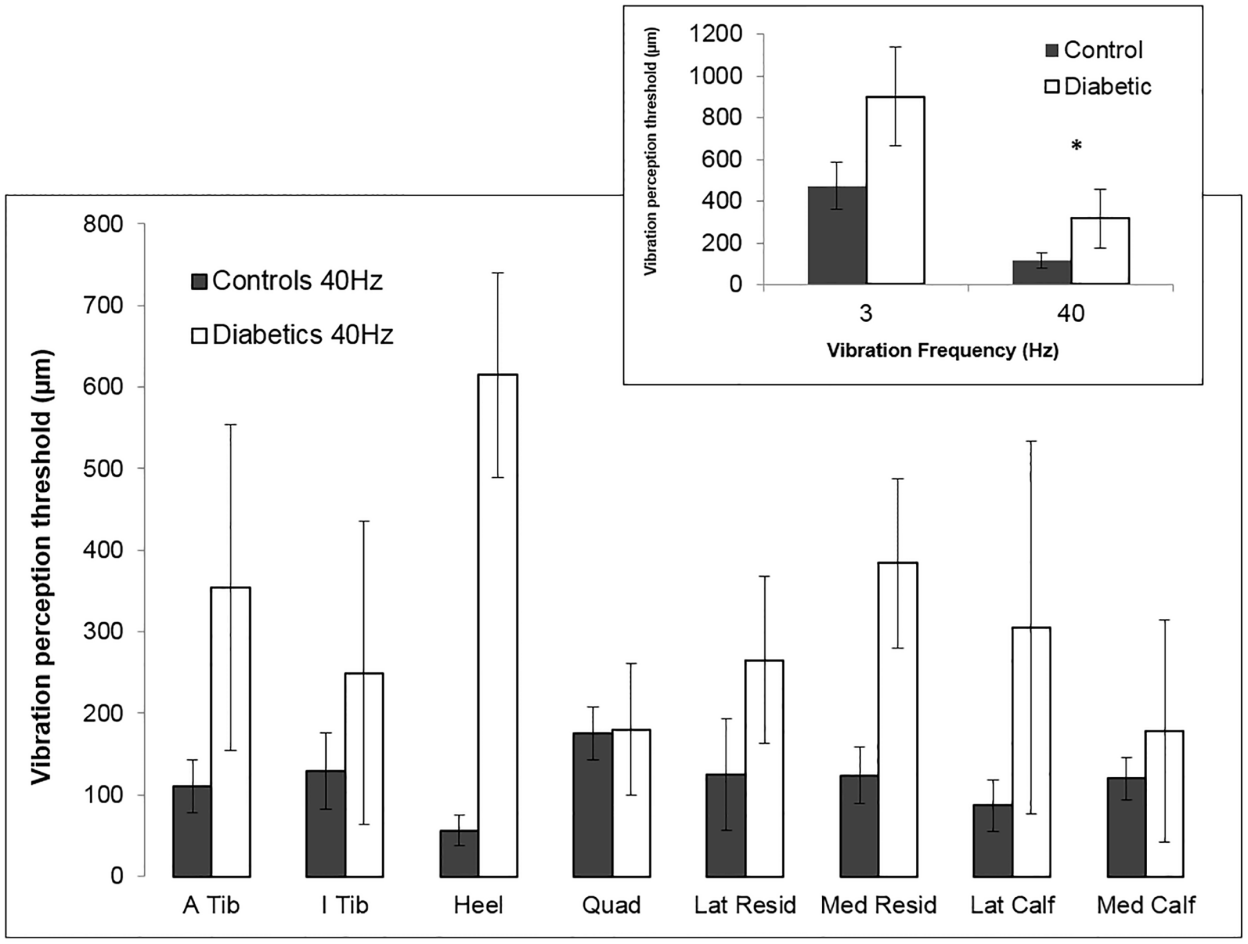
40Hz vibration perception threshold (VPT) (μm) of control (dark grey bars) and diabetic amputee (white bars) subjects across test sites. 40 Hz VPT is higher in diabetic amputees compared to controls for all sites except the quadriceps. INSET: At 40 Hz amputees with diabetes illustrated significantly higher thresholds compared to controls when averaged across sites 40 Hz (chi square < 0.0001). 3 Hz VPT followed a similar trend, however data were not analysed statistically due to ceiling effects. Error bars represent standard deviation.

**Fig 3:**
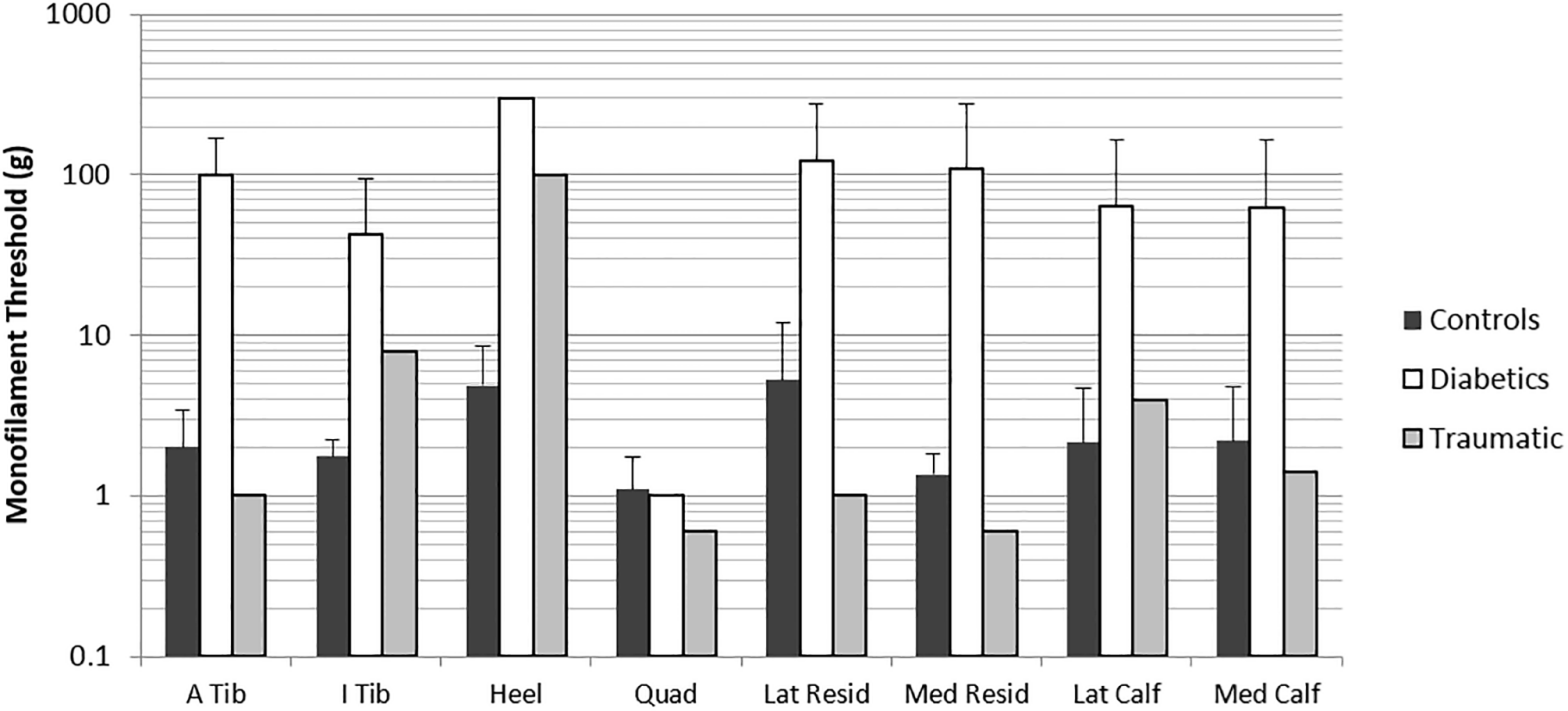
Comparison of control (dark grey bars), diabetic amputee (white bars) and traumatic amputee (light grey bars) groups for monofilament threshold. Diabetic amputees showed higher thresholds at all sites except for quadriceps when compared to controls. The traumatic amputee tended to show lower thresholds on the amputated limb compared to homologous sites on controls. Comparisons within group across site can also be made. The traumatic amputee shows higher thresholds on the intact limb (I tibia, medial calf, lateral calf) compared to the amputated limb (A tibia, med residual, lateral residual). Bars indicate mean values, error bars indicate standard deviation.

For all sensitivity measures (VPT and MF) amputees with diabetes showed markedly higher thresholds (lower sensitivities) compared to controls. Ceiling effects were present at 3 Hz and 250 Hz VPT and only 40 Hz VPT data were analysed statistically. 40 Hz thresholds were significantly higher in the group with diabetes compared to the control group (chi square < 0.0001; Fig 2). Although not analysed statistically (due to ceiling effects), the group with diabetes also showed higher thresholds for 3 Hz and 250 Hz VPT as well as MF when compared to controls (Table 1, Fig 3).

When examining site sensitivity differences within the group with diabetes, there was a trend in which thresholds decreased as sites moved more proximal. The highest thresholds were observed at the heel, and a decrease in threshold was observed as the sites moved more proximal with the lowest thresholds observed at the quadriceps (greatest sensitivity) (Fig 2). A strong trend was found for site, however, significant differences between sites were not found at 40 Hz, likely due to low n (p = 0.0588) (Fig 2). This trend was also apparent for 3 Hz VPT and MF data (Fig 3), but was not analysed statistically due to ceiling effects (Table 1). No major trends were observed when comparing sites on the amputated limb to homologous sites on the intact limb.

#### Traumatic amputee

**Fig 4:**
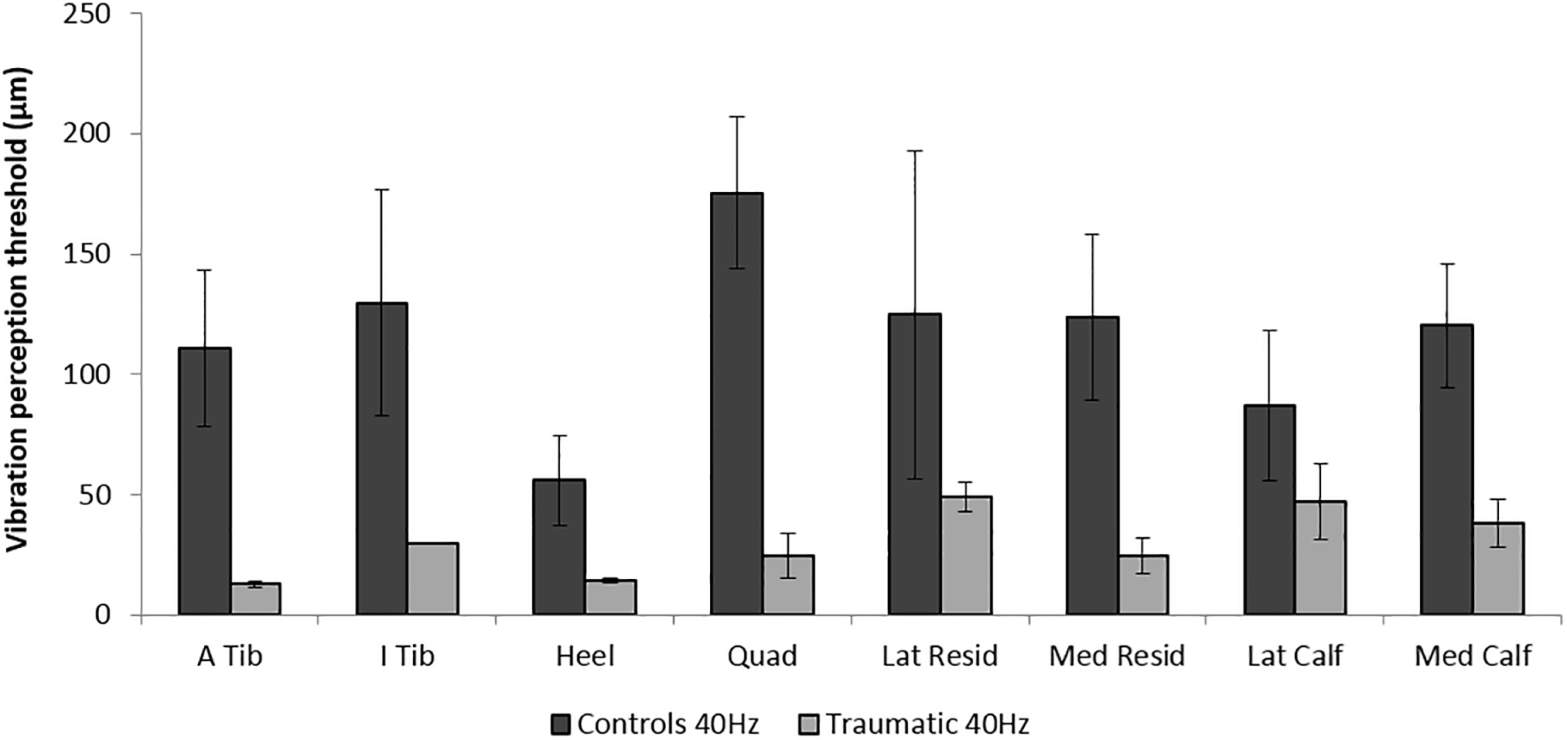
40Hz vibration perception thresholds (VPT) for control (dark grey bars) and traumatic amputee (light grey bars) subjects across all sites. At all sites the traumatic amputee had lower thresholds compared to control. Error bars indicate standard deviation. For control subjects, this standard deviation is calculated from the deviation between subjects. For the traumatic amputee, this standard deviation is calculated from the deviation between the 3 trials for that individual.

**Fig 5:**
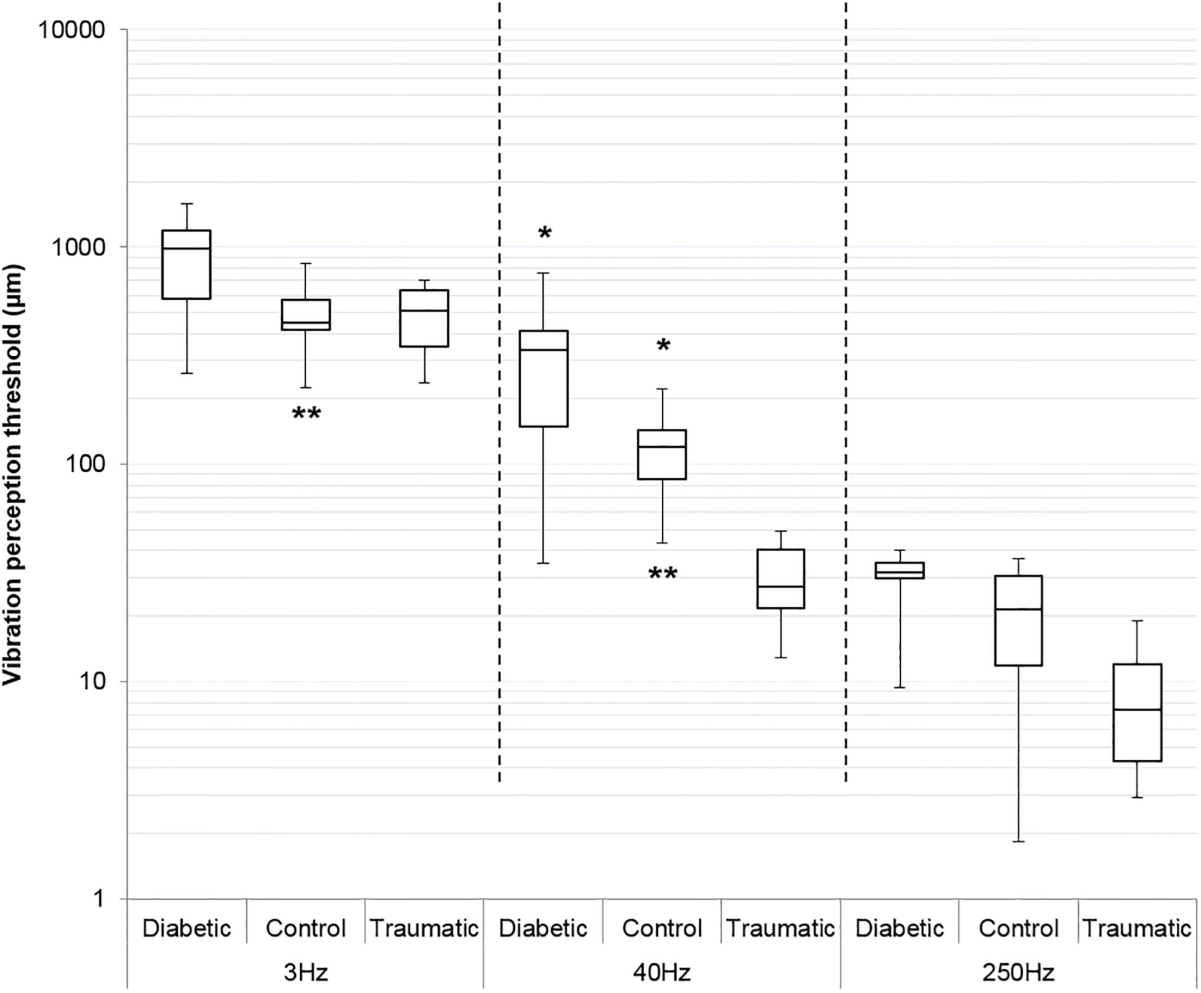
Box plot of vibration perception threshold (VPT) of amputees compared to controls at each frequency. Each box plot is based on all observations for that group at a given frequency collapsed across sites. Bar in middle of box represents median. Top and bottom of box are 75^th^ and 25^th^ quartiles respectively. Top whisker represents maximum value, bottom whisker represents minimum value. At 40 Hz, diabetic amputees show significantly higher threshold compared to controls (chi square<0.0001)*. Control subjects have significantly lower threshold at 40 Hz compared to 3 Hz (chi square<0.0001)**. Number of subjects for each group: control n = 4; diabetic n = 3; traumatic n = 1.

The traumatic amputee had lower thresholds compared to controls at both 40 Hz (Fig 4) and 250 Hz VPT (Fig 5). At the site of the amputated tibia, threshold sensitivity at 40 Hz was 86.6% lower (more sensitive) in the traumatic amputee compared to the homologous site on controls; in fact for all sites examined the 40 Hz VPTs were lower in the traumatic amputee compared to controls (Table 1). Even the smallest decrease in 40 Hz VPT threshold was substantial at 51.5% (Table 1). 250 Hz VPT were also markedly lower in the traumatic amputee compared to controls at all sites (Table 1, Fig 5). Interestingly, unlike controls who showed ceiling effects at 250 Hz, no ceiling effects were observed at any sites or any frequencies in the traumatic amputee (Table 1). No prevailing trend was apparent for MF or 3 Hz VPT between the traumatic amputee and controls.

### Relationship between skin sensation and functional balance

**Fig 6:**
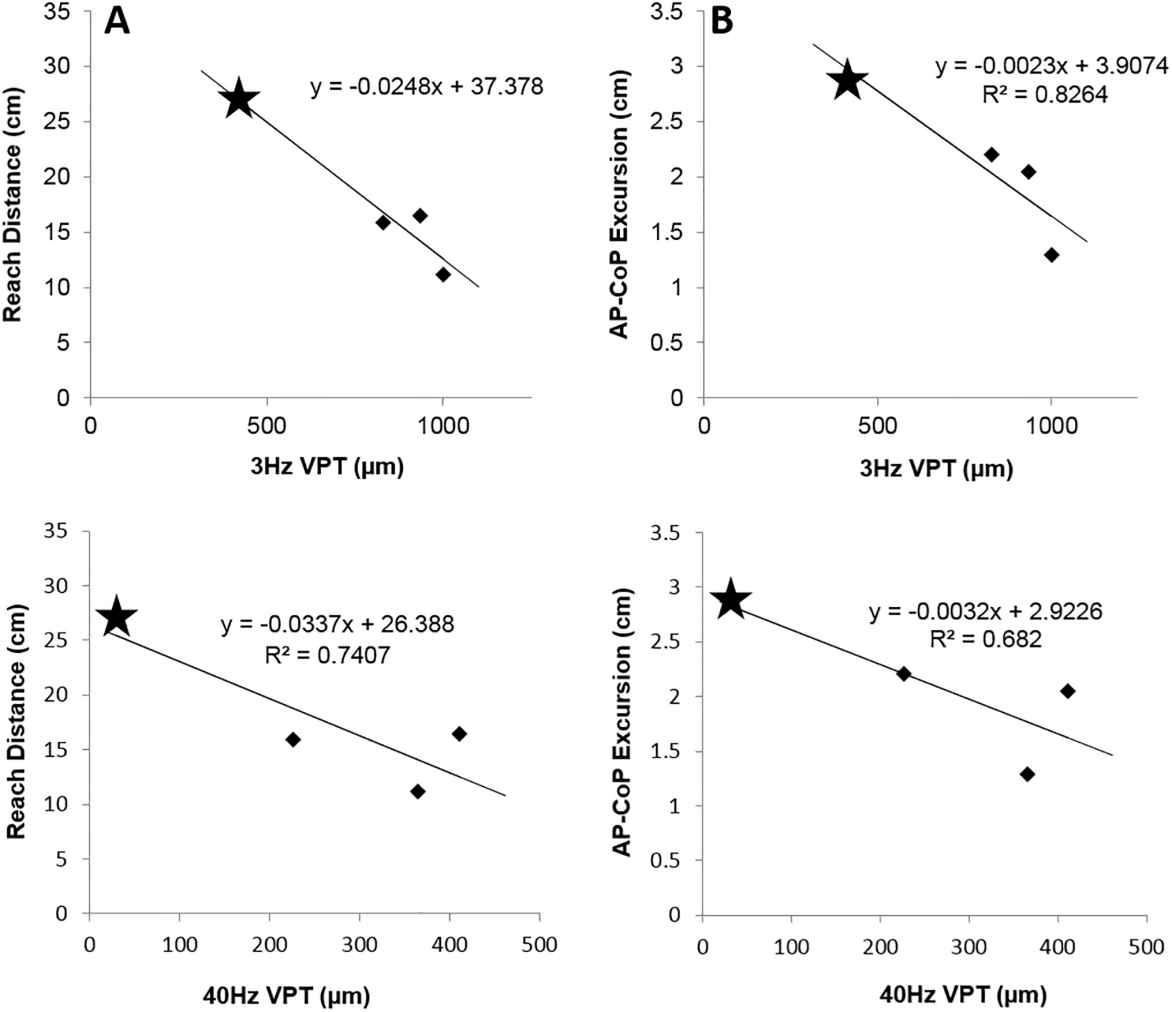
Correlations of amputee subject 3Hz and 40Hz VPT (for skin sites contacting the prosthetic) with reach distance (A and C) and CoP excursion (B and D) during functional reach test. The negative correlation shows subjects with high perception thresholds (low sensitivity) show shorter reach distance and decreased CoP excursion. The traumatic amputee is marked with star, and the diabetic amputees are marked with diamonds. Ceiling effects were observed for diabetic subjects at 3Hz VPT.

Average reach distance of the control subjects was significantly longer (32.5cm ± 8.6) than the amputees (17.7cm ± 6.7) and CoP excursion was also larger in control subjects (4.85cm ± 0.7) compared to amputee subjects (2.10cm ± 0.7). VPT for three skin sites within the prosthetic (amputated tibia, medial residual, lateral residual) were averaged (VPT prosthetic = VPT_p_) and correlated to measurements during functional reach. A negative correlation was observed between 3 Hz VPT_p_ and reach distance (r^2^ = 0.9413) and 3 Hz VPT_p_ versus CoP excursion (r^2^ = 0.8264) such that reach distance and CoP excursion decreased with increasing 3 Hz VPT_p_ (Fig 6A and 6B). Due to the 3 Hz VPT ceiling effects this correlational finding is limited. Although not statistically significant (p>0.05), 40 Hz VPT_p_ was also found to correlate with reach distance (r^2^ = 0.7407) and CoP excursion (r^2^ = 0.682) but without the confound of any ceiling effects (Fig 6C and 6D). Overall these data suggest that amputees with lower perception threshold perform better on functional measures of standing balance.

## Discussion

The current study examined cutaneous sensitivity in lower limb amputees using quantitative methods. As hypothesized, amputees with diabetes illustrated higher thresholds (lower sensitivity) to vibration at all frequencies compared to control subjects, likely due to diabetic peripheral neuropathy [32]. Interestingly, the one traumatic amputee tested showed an increase in sensitivity for VPT testing at both 40 Hz and 250 Hz. While no mechanisms were tested here, we discuss this finding below in the context of cortical reorganization following amputation, and address the functional implications of skin sensitivity. We found that amputees with higher skin sensitivity at locations that interact with the prosthetic performed better on the functional reach task, suggesting that skin in this region may provide important and useful information to the amputee in the control of balance and gait and may be a beneficial target for rehabilitation strategies. Limitations based on low sample size and observed ceiling effects have been considered throughout the paper, however we believe that the use of VPT to examine the role of different skin afferents illustrates the potential for sensitivity changes in amputees which has not been clearly shown in lower limb amputees previously.

### Amputees with diabetes

As hypothesized, amputees with diabetes had increased perception thresholds for VPT and MF compared to control subjects. This reduced sensitivity was evident bilaterally, and was statistically significant for 40 Hz VPT (chi square<0.05) with a trend observed at 3 Hz, 250 Hz and for MF. This is not a surprising result, considering the high incidence of peripheral neuropathy known to occur with diabetes [33]. The observation that neuropathy can be observed in the intact limb is consistent with the literature, however there has been limited quantitative measurement to date to examine how proximally the neuropathy extends. In one study that did examine progression of neuropathy, examination on the leg was limited to “normal” vs. “abnormal” sensation, rather than providing quantitative data [18]. Our results indicate that in diabetic amputees, neuropathy is present at least as high as 5 cm below the knee (location of tibial site) in both the amputated *and* intact limbs as compared to controls. Interestingly, the quadriceps site thresholds were relatively similar compared to controls, which suggests that despite the advanced stage of diabetes in these subjects, the neuropathy is restricted to the distal limbs. These findings support the use of MF and VPT testing to quantify disease progression in diabetic patients. Perception deficits were observed at all three frequencies, suggesting that the sensitivity of SAI, FAI and FAII afferents are all impacted by the disease.

A lack of adequate skin sensation is of concern in the prosthetic sites as they play an important role in identifying excessive pressure or chafing in the prosthetic. A reduced capacity to detect excessive pressures can lead to complications following amputation [34]. In addition, the skin within the prosthetic may serve a similar sensory role to the sole of the intact foot as an important source of sensory feedback during balance [1] and gait [35, 36]. Quai et al (2005) showed that impaired vibration perception (abnormal vs normal perception assessed with a tuning fork held against the skin) within the prosthetic of lower limb amputees was associated with increased CoP sway in quiet stance, and that poor vibration sense was a strong predictor of history of frequent falls in this population. Further, Kavounoudias et al (2005) found that a decrease in skin sensitivity may contribute to a decrease in kinesthesia at the knee joint that has been shown to occur in amputees with diabetes [8]. While we were unable to establish a significant correlation between sensitivity and balance measures (low n), a strong trend indicated that subjects who showed increased thresholds (lower sensitivity) within the prosthetic also exhibited a shorter reach distance and decreased CoP excursion. The strong correlation at 3 Hz is of particular interest as 3 Hz is believed to functionally target the SAI population of afferents that convey pressure information. 3 Hz VPT has previously been linked to balance performance [37] which suggests a role for pressure feedback in mediating the present reach distance measurements. During a leaning task such as the functional reach, adequate pressure perception, mediated by SAI afferents, may be vital in identifying how close the CoP traverses toward the boundary of the base of support. Therefore, amputee subjects with improved pressure perception within the prosthetic may be more comfortable reaching forwards and placing their CoP closer to the base of support boundary in this task. Unfortunately, these conclusions are limited by ceiling effects in diabetic subjects for 3Hz VPT. Correlations were not as strong for frequencies mediated by FAI afferents (40 Hz), although these were still in the moderate range (R^2^ = 0.6820 for AP-CoP and R^2^ = 0.7407 for reach distance). FAI afferents are also likely to be activated in the dynamic component of the functional reach task and would provide information about friction and slip within the prosthetic, which may explain the observed correlations. No significant correlations were shown at this frequency either (P>0.05). If in fact changes in tactile sensitivity can have a meaningful impact on functional balance, this provides an area of focus during rehabilitation; to restore as much afferent feedback from the amputated leg as possible through training or use of medication. Previous research has shown that the application of subthreshold [38, 39], and suprathreshold [40] foot sole vibrations can improve balance. Subthreshold vibrations are thought to leverage stochastic resonance lowering the firing threshold of the underlying mechanoreceptor afferents [39]; whereas step-synchronized suprathreshold vibrations may facilitate balance reflex pathways [40]. Future research should investigate if similar stimulation applied to the residual limb results in increased balance outcomes in lower limb amputees, as this may have implications for the design of feedback prosthetics, particularly those which attempt to provide tactile feedback to the user.

### Traumatic amputee

Compared to controls, the traumatic amputee showed much higher sensitivity to 40 Hz and 250 Hz vibration, and this was observed in both the intact and amputated limbs. These results may of course reflect individual variability; however, the traumatic amputee did not show increased sensitivity to monofilaments or 3 Hz compared to controls, suggesting a specific and not a general increase in sensitivity. While we cannot rule out the possibility that this individual may have had some factors influencing their peripheral sensitivity (skin mechanics, receptor density), this possibility seems remote. It has been suggested that individuals are born with a set number of mechanoreceptors, and it is likely there are a similar number of receptors across individuals [41, 42]. Peters et al, (2009) did show that decreases in surface area (for example small fingers) does increase tactile acuity as there is a greater density of receptors in the same anatomical region [41]. While we did not measure circumference of the residual limb, increased sensitivity of the traumatic amputee was also found on the intact limb, suggesting that it is likely not density driving the response. As such we feel we can rule out density of receptors as the cause of increased sensitivity, and rather support the notion that there is an increased influence of these remaining skin receptors on cortical firing and the observed increase in sensitivity may contribute to the hypothesis that peripheral sensitivity changes are a result of cortical reorganisation following amputation.

Amputation creates a rapid deafferentation that results in the associated brain regions being taken over by neighbouring cortical areas with these changes progressing over time [21,43]. Theoretically, this reorganisation results in an increased number of cortical neurons devoted to the residual limb, resulting in increased tactile acuity and sensitivity [44]. This model is supported by the observation that tactile acuity and sensitivity can be increased using an acute experimental decrease in afferent feedback (via anesthetic) to neighbouring areas [44,45]; which supports increased sensitivity observed in upper limb amputees [15,46]. In the current work, the increase in sensitivity of the skin within the prosthetic of the traumatic amputee may represent an adaptation to increase afferent feedback regarding the interaction between the prosthetic and the residual limb in an attempt to overcome the challenge of loss of feedback from the amputated foot sole. In addition, as the skin within the prosthetic becomes involved in weight bearing, more (and different) afferent signals will be sent to the brain than previously sent from that region of skin. Since it is known that increased afferent input can result in cortical expansion and reorganisation [47–49], this increase in afferent feedback from the residual limb may help strengthen the cortical reorganisation. Based on this hypothesis, it is reasonable to expect a similar increase in afferent feedback in the residual limb of diabetic amputees and subsequent increase in tactile sensitivity as a result of cortical reorganization. However increased sensitivity was not observed in diabetic amputees, which is likely due to the presence of significant peripheral neuropathy (reducing sensitivity) masking any central changes. In addition, the presence of peripheral neuropathy would reduce the number of afferent signals sent to the brain from the residual limb, and therefore may reduce the degree of cortical reorganization which takes place in this population.

The change in sensitivity at 40 Hz and 250 Hz (but not seen at 3 Hz) for the traumatic amputee suggests a change in tactile information mediated primarily fast adapting afferents. An up regulation of FAI and FAII mediated signals may be due to the functional relevance of these ‘dynamic’ afferents, which code for information regarding velocity of indentation and slips [22, 50]. Microneurographic recordings have shown FAI afferents to be the most prevalent afferent class in the glabrous skin of the foot sole, which highlights their importance in postural control [25, 51]. The observation that FAI mediated perception is up-regulated in the residual limb of the traumatic amputee suggests that feedback from the remaining areas of skin may adapt to better serve the function of the amputated foot sole, as the residual limb has now become the primary site of interaction between the amputee and the ground. In addition, the FAII afferents are specialized to detect high frequency vibration, and respond to distant stimuli, capable of sensing distant vibration through a tool, footwear or prosthetic [50].

Phantom limb sensation character and frequency were investigated using a subject questionnaire. The traumatic amputee described experiencing phantom sensations (presence, pain, burning and itching) very often (daily), whereas two diabetic amputees described phantom sensations approximately once per month, and one diabetic amputee described experiencing phantom sensations a few times per year. Based on work in upper limb amputees, Ramachandran and colleagues have suggested phantom sensations to be a kind of perceptual correlate of cortical reorganization. It is thought that as afferent feedback from the surrounding area “takes over” the brain areas previously corresponding to the amputated limb, and spontaneous discharge of neurons in this area is misinterpreted as originating from the missing limb, causing a phantom sensation [52]. This hypothesis was strengthened by previous work showing a correlation between cortical reorganization and perceived magnitude of phantom limb pain [17,53]. However, recent work by Makin et al. (2015) has challenged the role of cortical reorganization of the somatosensory cortex in phantom limb sensations in upper limb amputees [54]. This work has shown the extent of cortical reorganization in upper limb amputees may be smaller than previously suggested, and that there is no correlation with phantom limb sensations, as has been previously shown. From our data we cannot conclude if the phantom sensations are related to the magnitude of cortical reorganization in the traumatic amputee. Although not shown in the current study, there is potential that the use of afferent feedback of the residual limb for balance and gait may increase the amount of cortical reorganization which occurs in lower limb amputees compared to upper limb amputees. In addition, the mechanisms which may mediate changes in tactile sensation rather than phantom limb sensations following amputation should be investigated further.

### Conclusion

As expected, tactile sensation was significantly lower in amputees with diabetes compared to control subjects, however this decrease in sensitivity was limited to the distal limb. Due to the role of the skin of the foot sole in balance and gait, increasing tactile feedback from skin which interacts with the prosthetic may improve functional balance outcomes. Interestingly, the traumatic amputee showed increased skin sensitivity relative to controls and also showed the best performance on the functional reach test. While not confirmed with neuroimaging, this increased sensitivity may be due to cortical reorganisation which is known to occur following amputation.

## Supporting information

S1 Text Supporting information vibration data. (XLSX)

S2 Text Supporting information monofilament data. (XLSX)

S3 Text Supporting information reach data. (XLSX)
